# Pathologic evolution-related Gene Analysis based on both single-cell and bulk transcriptomics in Colorectal Cancer

**DOI:** 10.7150/jca.49262

**Published:** 2020-10-22

**Authors:** Jiali Li, Zihang Zeng, Jiarui Chen, Xingyu Liu, Xueping Jiang, Wenjie Sun, Yuan Luo, Jiangbo Ren, Yan Gong, Conghua Xie

**Affiliations:** 1 Department of Radiation and Medical Oncology, Zhongnan Hospital of Wuhan University, Wuhan, China.; 2 Department of Biological Repositories, Zhongnan Hospital of Wuhan University, Wuhan, China.; 3 Human Genetics Resource Preservation Center of Hubei Province, Zhongnan Hospital of Wuhan University, Wuhan, China.; 4 Hubei Key Laboratory of Tumor Biological Behaviors, Zhongnan Hospital of Wuhan University, Wuhan, China.; 5 Hubei Cancer Clinical Study Center, Zhongnan Hospital of Wuhan University, Wuhan, China.

**Keywords:** single-cell sequencing, colorectal cancer, pathologic stage, prognosis, TCGA, GEO

## Abstract

**Purpose:** The patients diagnosed with colorectal cancer (CRC) are likely to undergo differential outcomes in clinical survival owing to different pathologic stages. However, signatures in association with pathologic evolution and CRC prognosis are not clearly defined. This study aimed to identify pathologic evolution-related genes in CRC based on both single-cell and bulk transcriptomics.

**Patients and methods:** The CRC single-cell transcriptomic dataset (GSE81861, n=590) with clinical information and tumor microenvironmental tissues was collected to identify the pathologic evolution-related genes. The colonic adenocarcinoma and rectum adenocarcinoma transcriptomics from The Cancer Genome Atlas were obtained as the training dataset (n=363) and 5 other CRC transcriptomics cohorts from Gene Expression Omnibus (n=1031) were acquired as validation data. Graph-based clustering analysis algorithm was applied to identify pathologic evolution-related cell populations. Pseudotime analysis was performed to construct the trajectory plot of pathologic evolution and to define hub genes in the evolution process. Cell-type identification by estimating relative subsets of RNA transcripts was then executed to build a novel cell infiltration classifier. The prediction efficacy of this classifier was validated in bulk transcriptomic datasets.

**Results:** Epithelial and T cells were elucidated to be related to the pathologic stages in CRC tissues. Pseudotime analysis and survival analysis indicated that HOXC5, HOXC8 and BMP5 were the marker genes in pathologic evolution process. Our cell infiltration classifier exhibited excellent forecast efficacy in predicting pathologic stages and prognosis of CRC patients.

**Conclusion:** We identified pathologic evolution-related genes in single-cell transcriptomic and proposed a novel specific cell infiltration classifier to forecast the prognosis of CRC patients based on pathologic stage-related hub genes HOXC6, HOXC8 and BMP5.

## Introduction

Colorectal cancer (CRC) results in as many as 900,000 deaths each year, accounting for approximately 9.2% cancer mortality worldwide 1. It is expected to increase by more than 20% to 1,100,000 in 2030 2. In addition, statistical analysis indicated that its incidence substantially increased in patients younger than 40s 3, 4. Although the developments of early diagnosis, immunotherapy and chemotherapy remarkably facilitate the detection and curation of CRC, the overall survival remains less than 60% in developed countries 5. During the last few decades, numerous researches focused on molecular heterogeneity in colorectal oncocytes 6, 7, which probably contributed to totally divergent clinical endings. Explorations on the underlying genetic alteration are highly required to benefit CRC patients.

The pathologic stage exerts a decisive role in different therapy strategies as well as clinical treatment outcomes 8-10. However, the signatures of pathologic progress and stage promotion remain unclear. Surgery is recommended as the preferred treatment in stage I colon cancer, and chemotherapy composes of the therapeutical strategy as an integral part for patients at III or IV stage 5. Hence, more prognostic markers are required to uncover the decisive genes in pathologic processes, like RAS activation or function loss of TP53 during CRC tumorigenesis 6. Moreover, with the advances in single-cell RNA sequencing (scRNA-Seq) technique, a novel approach is provided to clarify the strong heterogeneity in predominant cell populations and profound alteration of key gene expression 11, 12. As is reported, CRC is classified into 4 subtypes based on consensus molecular subtypes (CMS) 7. The pathways or genes implicated in each subtype are unique: hypermutated, strong immune activation and microsatellite unstable in CMS1; striking WNT and MYC pathway activation in epithelial in CMS2; marked metabolic dysregulation of epithelial in CMS3; overt transforming growth factor-β (TGF-β) activation, stromal angiogenesis and invasion within mesenchymal in CMS4. In brief, dominant gene expression alteration in decisive cell populations attracted increasing attention.

In this study, we combined scRNA-seq and bulk transcriptomic to identify pathology-related cell populations and pathologic progress-related hub genes. A novel specific cell infiltration classifier was established to forecast pathologic stages and prognosis of CRC patients based on the 3 hub genes HOXC6, HOXC8 and BMP5. Our study provided a potential classification and key biomarkers to screen out CRC patients with primary pathology stages that were more likely to obtain a better prognosis.

## Material and Methods

### Data Preprocessing and Quality Control

**Figure 1:**
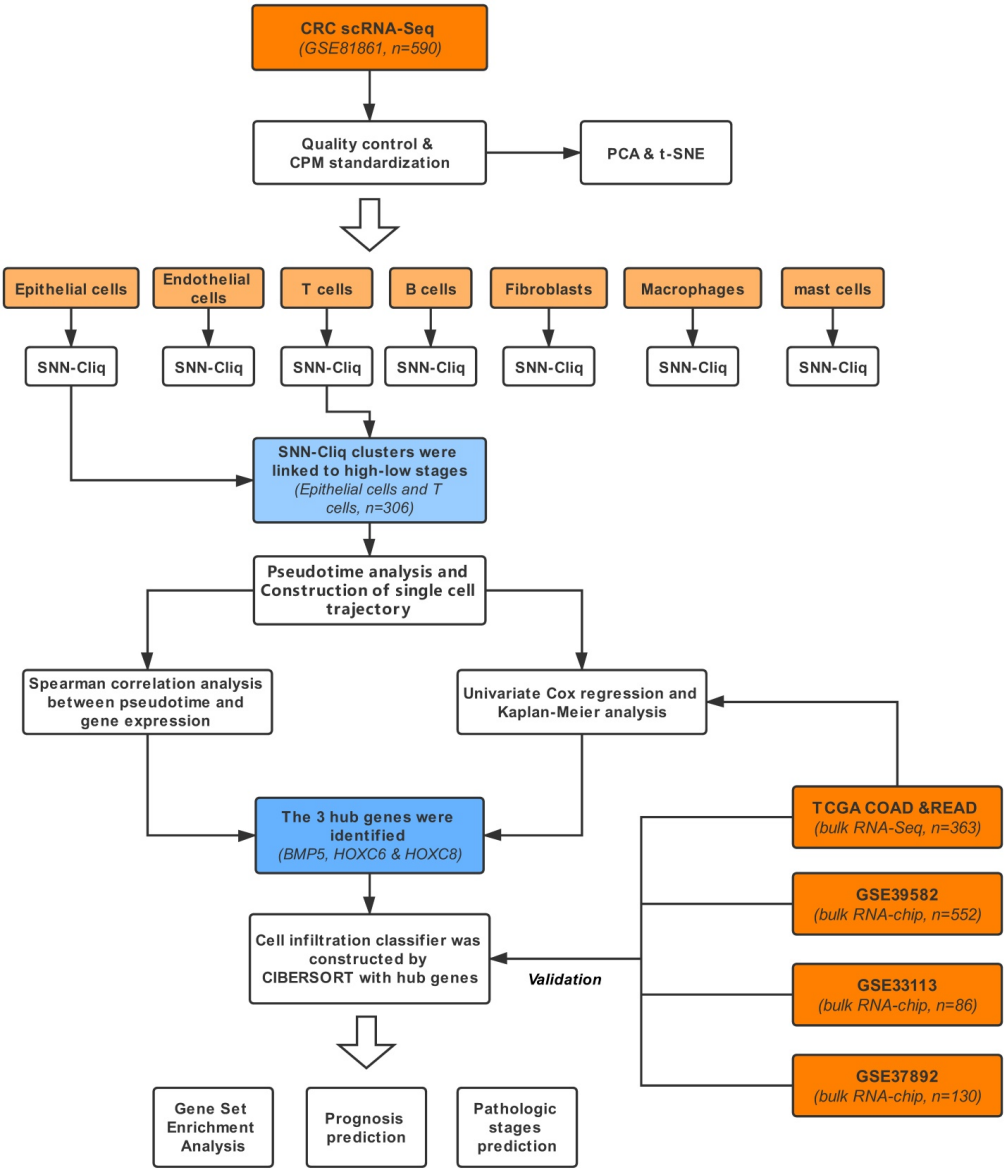
Workflow of this study.

The workflow is exhibited in Figure 1. The dataset (GSE81861) consisting of scRNA-Seq and clinical information of 11 primary CRC patients 13 was obtained from Gene Expression Omnibus (GEO) database. A total of 590 single-cell samples derived from CRC tissues or matched normal mucosa were included in this study. The selected cell samples contained different cell populations, including epithelial cells (tumor cells), endothelial cells, T cells, B cells, fibroblasts, macrophages and mast cells. Only cells that possessed at least 10,000 total counts and 500 expressed genes were included in the following analysis. The cell-level and sequencing profile diagnosis were executed sequentially. At the same time, count-per-million standardization was applied to optimize the library size. Quality control of scRNA-Seq was conducted with *scater* package.

Bulk RNA sequencing (RNA-Seq) of CRC cohorts were acquired as independent training dataset including colonic adenocarcinoma (COAD) and rectum adenocarcinoma (READ) data from the cancer genome atlas (TCGA), containing 363 CRC patients, Transcripts Per Million (TPM) standardization method and Z-score normalization were subsequently performed on TCGA RNA-Seq. Validation datasets were bulk genome chip data acquired from GEO database (GSE39582, n=552; GSE37892, n=130; GSE12945, n=62; GSE17537, n=55; GSE17538, n=232) 14-16. All the above 5 datasets were processed with robust multi-array average (RMA) standardization and Z-score normalization. The outlines of the datasets were displayed in Table 1, including sample capacity, age, gender proportion and survival. The clinical characteristic used in this study was the pathologic stage, which was assessed based on the standard of American Joint Committee on cancer. Stages higher than 2 (including pathologic stage 2) were regarded as high pathologic stages and stage1 was defined as low pathologic stages.

### Dimensionality Reduction

Principal components analysis (PCA) was a classical linear dimensionality reduction algorithm 17, 18. Eigenvalues and eigenvectors of covariance matrix were calculated to estimate correlations between variables. After that, several larger eigenvectors were extracted from the matrix represented as the principal components.

T-distributed stochastic neighbor embedding (t-SNE) was a nonlinear dimensionality reduction algorithm 19 used to dispose of the nonlinear correlations between variables. In the process of dimensionality reduction, instead of Euclidean distance, this algorithm selected conditional probability of choosing another point as the adjacent node to reflect the similarity of 2 nodes 20. For strict quality estimation, both t-SNE and PCA were applied to investigate the distribution of cell types and tissue types with the log-transformed expression values in R software.

### Graph-Based Clustering Analysis

A novel algorithm named shared nearest neighbor (SNN)-Cliq was developed combining SNN method with quasi-clique-based clustering method 21. In SNN-Cliq, input nodes were the vectors of gene expression within an individual cell. Similarity (Euclidean distance) between points was utilized as a weighted edge to construct SNN graph. In addition, graph-theoretic techniques were included to cluster the sparse SNN graph 22. This algorithm not only included Euclidean distance as a similarity measure but also combined with a quasi-clique-based clustering algorithm to accurately identify the highly similar nodes in the same cluster. Graph-based clustering analysis was implemented to subclassify the pathologic stage-related cell populations with *scran* package in R.

### Pseudotime Analysis

Pseudotime analysis was an algorithm to construct the development trajectory of a single-cell lineage according to the gene expression profile 23. It infers the cell development trajectory from the expression level changes of the pre-defined phenotype-related gene list and then distributes every single cell with its proper pseudotime in this trajectory. Cell development processes were exhibited on the trajectory plot. The above process was realized by *monocle* package using R to restore the development trajectory of pathologic evolution-related cell populations.

### Gene Set Enrichment Analysis

Gene set enrichment analysis (GSEA) was a function annotation approach aiming to identify probable significant biologic expression-changed signatures based on known gene sets 24. The enrichment score was calculated based on whether a certain gene belonged to the known gene sets or not. P values and normalized enrichment scores of certain pathways were obtained via permutation test. GSEA was achieved by *clusterProfiler* package to elucidate the key genes among differential pathologic evolution-related cell population clusters 25.

### Cell Infiltration Abundance Estimation

Cell-type identification by estimating relative subsets of RNA transcripts (CIBERSORT) was a computational deconvolution approach 26 to characterize cell composition of a mixture based on bulk gene expression profiles. The expression matrix of pre-defined cell markers worked as the reference to estimate the relative proportion of specific cell types in bulk RNA-seq profiles. A linear support vector regression, a machine learning method to denoise, was applied to deconvolve the bulk gene expression matrix. To establish the specific cell infiltration classifier, a gene matrix defining 2 different populations (C1 and C2 populations) was created using expression quartiles of the pathologic hub genes in CRC bulk transcriptome and then was input as the reference matrix, representing for 2 pathologic evolution-related cell classifications. The concrete process was performed using *CIBERSORT* package in R.

### Statistical Analysis

To identify the hub genes and validate the novel cell infiltration classifier, survival analysis was performed using both Kaplan-Meier survival estimation for classified variables, and Cox proportional hazards regression for quantitative index by R *survival* package. Chi-square test was implemented to verify the relevance between pathologic evolution-related cell classifications and realistic pathology stages. Spearman and Pearson correlation analysis were calculated respectively via the stats package. The above statistical analyses were completed with R 3.6.1. In all hypothesis tests, *P*-values less than 0.05 were regarded as statistical significance. All the *P*-values were two-sided.

## Results

### Quality estimation of scRNA-Seq

**Figure 2:**
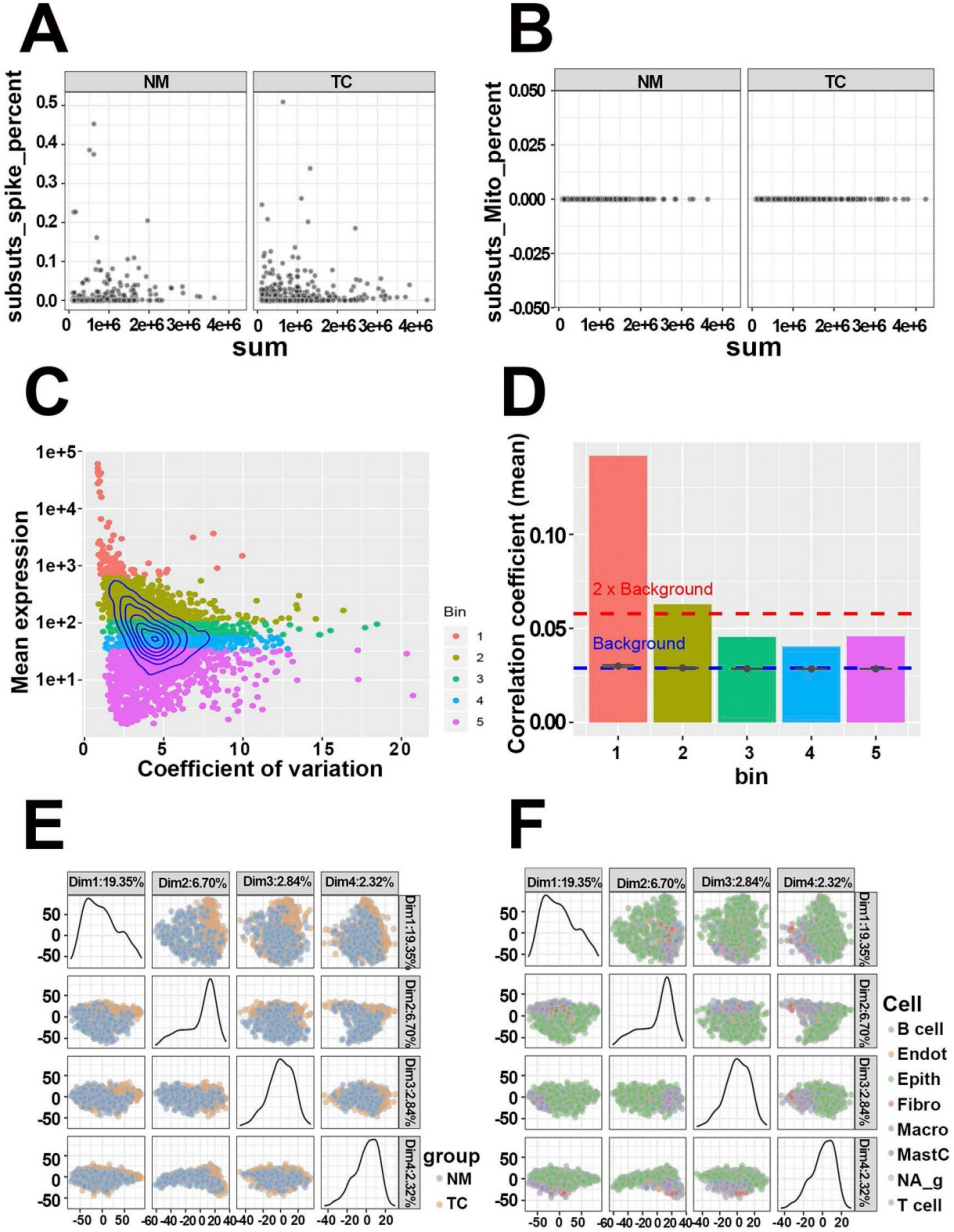
Quality control of single-cell RNA-Seq from GSE81861 dataset. (**A**) Respective detection of ERCC spike-in genes as control features in tumors tissues and matched normal mucosa. (**B**) Respective detection of mitochondria genes as control features in tumors tissues and matched normal mucosa. (**C**) Topographic map of features expression. The relationships between gene mean expression and coefficient of variation were displayed in this map with 5 different colors bins representing for corresponding expression levels. (**D**) Histogram of correlation coefficients of each bin calculated by Pearson correlation. Every feature in each bin was correlated to every other feature in the same bin. Mean value of the correlations were taken as the vertical axis variable. (**E**) Principal components extracted from covariance matrix between colorectal tumor tissue and normal mucosa. (**F**) Principal components extracted from covariance matrix between different cell populations.

The quality of GSE81861 containing 590 single-cell samples from 11 primary CRC patients with tumor and microenvironmental cell populations were first evaluated. The ERCC spike-in and mitochondria genes serving as known control signatures were detected in each cell to calculate the percentage of counts that come from the feature control set (Figure 2A, Figure 2B). Well-behaved cells, which contained a large proportion of expressed features as well as a small ratio expression of spike-in and mitochondria features, while others were discarded. At the same time, the mean expressions of features and coefficient of variation in cells were calculated to acquire highly expressed features (Figure 2C). Five bins with similar expression levels were encapsulated and shown in Figure 2D. Genes in bin 3, 4, and 5 that fell below the 2 * background were excluded. The filtered 1261 genes and 590 cells with high quality were preserved to the next step. Dimensional reduction results implied that cells from CRC tissues and normal mucosa were distinctly separated (PCA, Figure 2E; t-SNE, Figure S1A). The same results were observed in cell populations (PCA, Figure 2F; t-SNE, Figure S1B). The quality estimation outcome demonstrated that GSE81861 scRNA-Seq had a good performance in the strict process of quality control.

### Identification of pathologic stage-related cell populations

We selected 375 CRC cells with pathologic stage information of patients for downstream analyses. First, each cell type was clustered using graph-based methods. Tumor epithelial cells were classified into 6 clusters (Table S1), which distinguished high and low stages (*P*<0.0001, Fisher exact test). At the same time, T cells were sorted into 2 clusters respectively (Table S2). As expected, the 2 clusters completely corresponded to different stages (*P*<0.0001, Fisher exact test). However, other cell populations, including endothelial cells, B cells, fibroblasts, macrophages and mast cells exhibited no statistically distinct association with pathologic stages (all, *P*>0.05, Fisher exact test).

To gain better insight into signature expression in heterogeneous cell clusters, we detected maker genes of each pathologic stage-related cell cluster in T cells and tumor epithelial cells. Several signatures were significantly elevated in the clusters of epithelial cells with a low pathologic stage (Table S3): tumor necrosis factor receptor superfamily member 11b and kallikrein-related peptidase 7 in cluster 1, cadherin related family member 2 in cluster 5, growth factor receptor-bound protein 14, lymphoid enhancer-binding factor 1 and dynamin 1 in cluster 6. Within the cluster of T cells related to low pathologic stages, C-C chemokine receptor type 8, CD27 molecule, cell division cycle 34 and cyclin-dependent kinase 4 in cluster 2 were overexpressed compared to high pathologic stage-related cluster 1 (Table S4).

### Definition of pathologic stage evolution-related hub genes

**Figure 3:**
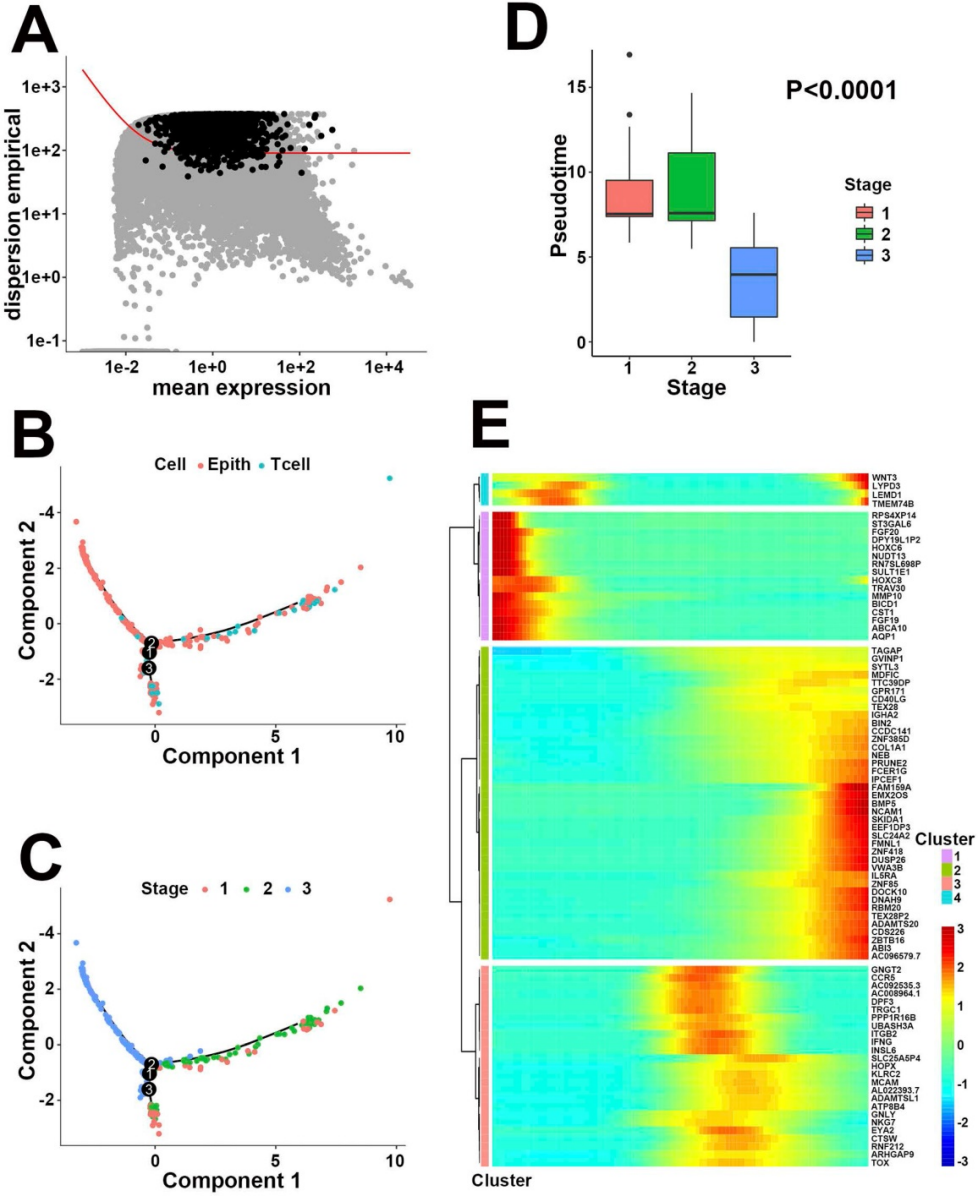
Negative correlation between pseudotime and pathologic stage. (**A**) The identification of 2356 differentially expressed features based on the average expression level and unusually variable expression across cells. (**B**) Trajectory analysis colored by cell types. (**C**) Trajectory analysis colored by pathologic stages. Stage 3 was located far distant from stage 1 and 2. (**D**) Box plot between pseudotime and stage. Stages 1 and 2 showed similar pseudotime levels while stage 3 exhibited lower pseudotime compared to other stages. (**E**) Heatmap of gene expression level varying with pseudotime in 4 clusters. Enhanced genes (in red) with lower pseudotime in cluster 1 represented for the higher pathologic stages, while upregulated signatures (in red) in cluster 2 performed completely the opposite.

To identify pathologic stage evolution-related hub genes, we next applied pseudotime analysis to the 306 cells that belonged to either epithelial or T cells in scRNA-Seq. A total of 2356 differentially expressed genes in response to pathologic stage evolution process were filtered based on the average expression level and dispersion empirical across cells (Figure 3A). The inferred developmental trajectory was demonstrated as a tree-like structure, exhibiting different cell states and gene expressions (Figure 3B). Pathologic stages presented a divergent distinction between high and low stages, with stage 3 located distant from stages 1 and 2 in trajectory plot (Figure 3C). The box plot between pseudotime and stages supported our speculation that pathologic stage classification was negatively correlated with pseudotime (Figure 3D, *P*<0.0001, variance analysis). On the other hand, Spearman correlation analysis was applied to 2356 differentially expressed genes to determine the potential pseudotime-related signatures. According to above analyses, a total of 64 pathologic stage positive-related genes and 20 negative-related genes were extracted from the single-cell expression profile (Table S5, *P*<0.05, |R|>0.2). Heatmap visualized all the pseudotime-dependent genes into 4 clusters based on their pseudotemporal expression pattern (Figure 3E). Taken together, our results indirectly linked pathologic stage to gene expression bridged by pseudotime, screening out the 84 pathologic stage evolution-related genes at a single-cell level. Subsequently, both univariate cox proportional hazards regression and Kaplan-Meier survival estimation were executed to assess the prognostic effect of the 84 genes on primary CRC patients from the TCGA bulk transcriptomics database. HOXC6, HOXC8 and BMP5 exhibited statistical significance on prognosis prediction in both methods (all, *P*<0.05, Table 2). Based on the aforementioned process, the 3 hub genes with correlation to both pathologic evolution and prognosis in CRC were finally determined.

**Figure 4:**
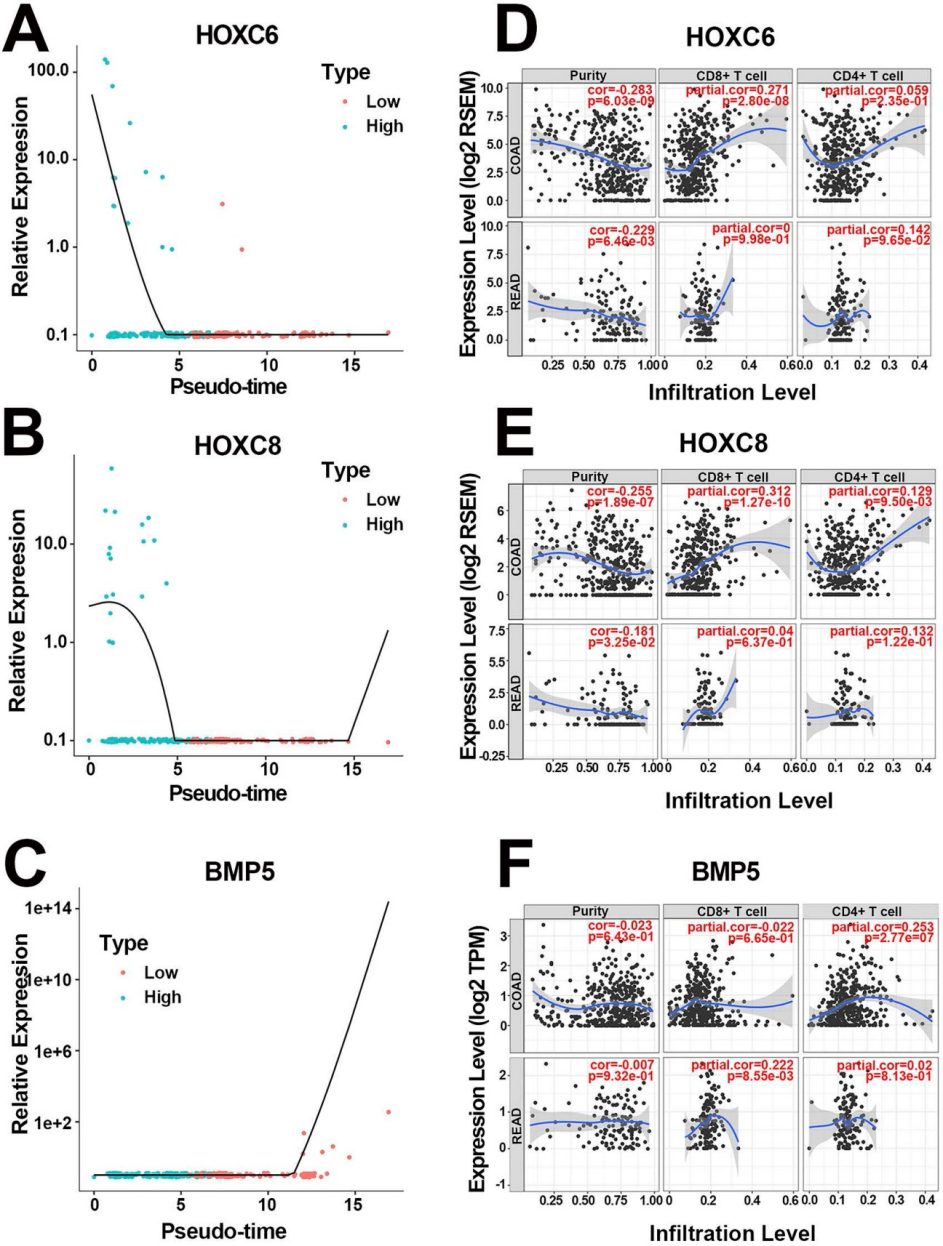
Portraits of the 3 hub genes. (**A**) The pseudotime-expression scatter diagrams of HOXC6. Cells with higher pathologic stage possessed smaller pseudotime and higher HOXC6 expression level. (**B**) The pseudotime-expression scatter diagrams of HOXC8. Cells with higher pathologic stage possessed smaller pseudotime and higher HOXC8 expression level. (**C**) The pseudotime-expression scatter diagrams of BMP5. Cells with higher pathologic stage possessed smaller pseudotime and lower BMP5 expression level. (**D**) Immunocyte infiltration map of HOXC6 in COAD and READ. (**E**) Immunocyte infiltration map of HOXC8 in COAD and READ. (**F**) Immunocyte infiltration map of BMP5 in COAD and READ.

More specifically, the scatter diagrams separately depicted the distribution of the 3 hub genes in high and low pathologic stages, varying with pseudotime in scRNA-Seq (Figure 4A-C). The results confirmed that highly expressed HOXC6 and HOXC8 as well as downregulated BMP5 were associated with higher pathologic stages. In addition, survival analysis suggested that both HOXC6 and HOXC8 were associated with poor prognosis (both HR=1.90, P<0.005, Table 2), whereas BMP5 played a positive role in better clinical outcome (HR=0.57, P=0.012, Table 2). Mean expression value calculation indicated that HOXC6 and HOXC8 were expressed only in epithelial cells, while BMP5 was activated in both cell populations (Figure S2A). The detailed expression levels of the 3 hub genes in different cell populations were displayed in Figure S2B-D. Moreover, correlation analysis validated the statistically significant relationship between pseudotime and expression levels again, separately in both cell populations (Table 3). Finally, T cell immune infiltration portraits of hub genes in COAD and READ were explored in Tumor Immune Estimation Resource database 27 (Figure 4D-F).

### Cell infiltration classifier construction, evaluation and validation

**Figure 5:**
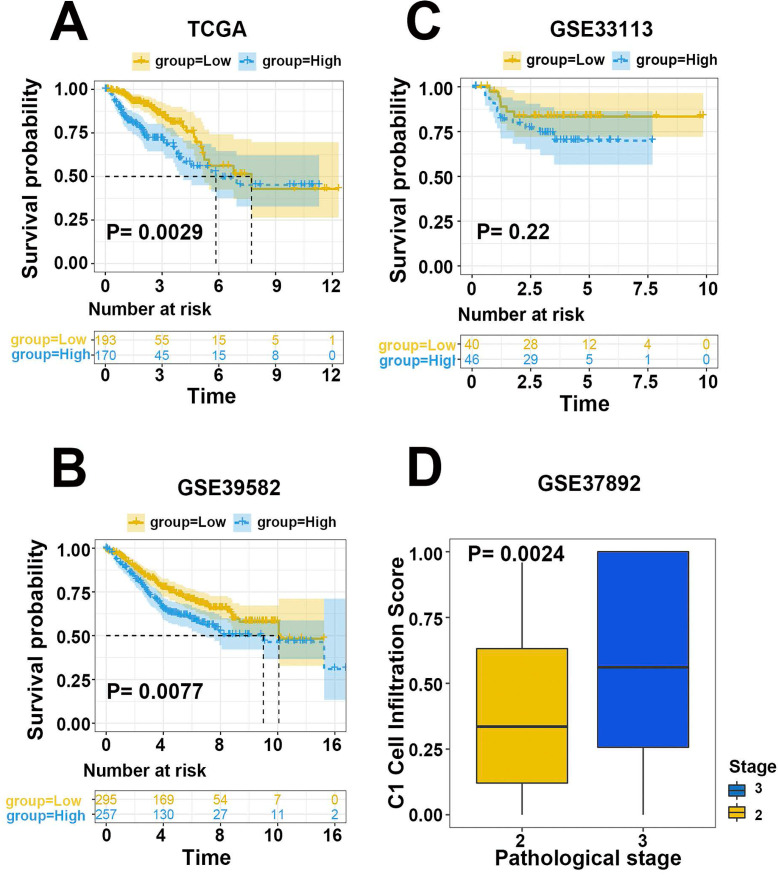
Evaluation and validation of efficiency of the cell infiltration classifier on prognosis.(**A**) Prognosis analysis with TCGA CRC training dataset. Patients in low C1 cells infiltration group had significantly better survival than those in the high group. (**B-D**) Prognosis analysis with independent validation datasets GSE39582, GSE12945 and GSE17538.

According to the differential expression pattern of hub genes, we defined 2 cell classifications as the reference matrix. C1 population represented a cell category, in which HOXC6 and HOXC8 were highly expressed, while BMP5 was downregulated while C2 cells behaved oppositely (Table S6). Based on the support vector regression algorithm, CIBERSORT accurately calculated the relative proportions of distinct cell classifications in TCGA CRC bulk transcriptomics (Table S7). To confirm the efficiency of the 2 cell classifications on prognosis prediction, survival analysis was applied to these patients. Patients with C1/C2 cell infiltration coefficients larger than 1 were considered as high C1 infiltration group, and results suggested that patients in higher C1/C2 ratio group suffered from a significantly unfavorable survival (Figure 5A, *P*=0.0029). Meanwhile, the outcome of univariate Cox regression with the same TCGA dataset supported the above conclusion (*P*=0.002). Correlation analysis implied that the lower C1/C2 ratio exhibited a statistical correlation with the lower pathologic stage in CRC patients (*P*=0.028, Spearman correlation test). Of note, all the 3 hub genes were involved in the TGF-β signaling pathway. GSEA results suggested that cellular response and regulation to TGF-β stimulus contributed to the distinct clinical results of the 2 infiltration groups (Table S8). Thus, a practical cell infiltration classifier based on the expression level of HOXC6, HOXC8 and BMP5 was eventually established to predict prognosis, as well as pathologic stage differences in primary CRC patients.

**Figure 6:**
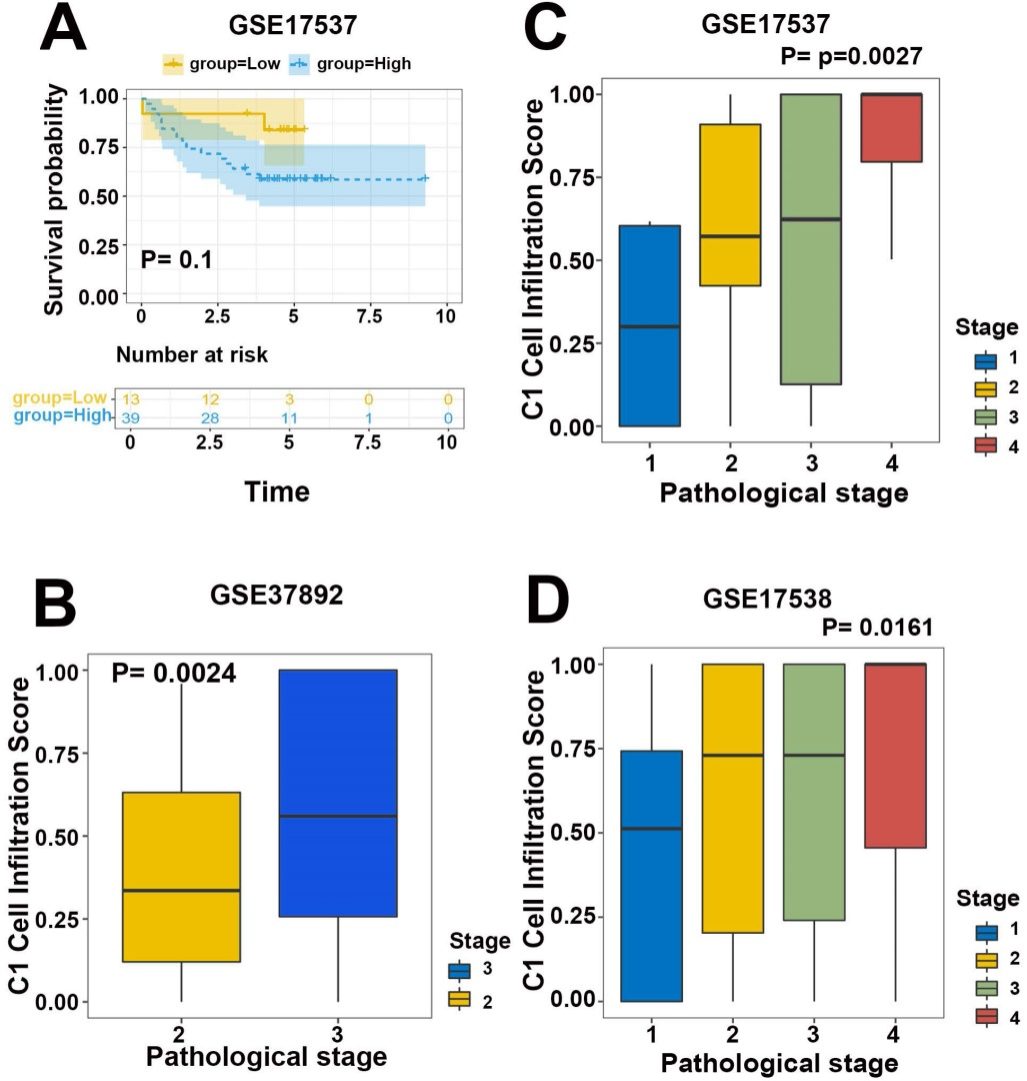
Validation of efficiency of the cell infiltration classifier on prognosis and pathologic stages. (**A**) Patients in low C1 cells infiltration group in GSE17537 had better survival tendency. (**B-D**) Box plot between pathologic stage and C1 cell infiltration with independent validation datasets GSE37892, GSE17537 and GSE17538. Lower pathologic stages were associated with lower C1 cell infiltration scores.

To validate the efficiency of our cell infiltration classifier, a set of independent primary CRC bulk genome chip datasets (GSE39582, GSE33113, GSE37892, GSE12945, GSE17537, and GSE17538) was collected from the GEO database. The 3 hub gene expression profiles were relatively extracted out as the input data of our classifier and the cell infiltration estimation classifications of each patient were defined based on C1/C2 proportion. Subsequent survival analysis exhibited the lower C1/C2 proportion were statistically linked to better prognosis of CRC patients (HR= 1.48, *P*=0.0077, GSE39582; HR=3.36, *P*=0.096, GSE12945; HR= 1.52, *P*=0.072, GSE17538; HR= 3.16, *P*=0.1, GSE17537; Figure 5B-D, Figure 6A). Variance analysis was applied to the sample to validate the association between the infiltration C1/C2 ratio and pathologic stages. Box plot implied lower pathologic stages were associated with lower C1/C2 ratio (*P*=0.0024; *P*=0.0027; *P*=0.016; Figure 6B-D). These results validated the prediction efficiency of our novel constructed cell infiltration classifier based on the expression of the 3 hub genes, confirming that CRC patients with upregulated BMP5 and downregulated HOXC6/8 were related with lower pathologic stages and better prognosis.

## Discussion

With the iteration of cell sequencing technology, more details about differentiation map and transcriptional heterogeneity have been clarified 28, 29. The application of scRNA-Seq facilitates the excavation of tumorigenesis and molecular classifications compared to bulk sequencing 30-32. By combining scRNA-Seq with clinical characteristics, we revealed the hub genes in the course of CRC pathologic stage evolution and clarified the impact of differential cell infiltration classifications on CRC prognosis based on hub gene expression. A novel classifier to estimate specific cell infiltration was established, which might indicate the pathologic stages and clinical outcomes.

Based on the application of scRNA-Seq, 2 distinct subtypes of cancer-associated fibroblasts associated with prognosis and an mRNA-miRNA regulatory network of CRC were identified in previous studies 13, 33. However, in this study, tumor epithelial and T cells were discovered to exert an essential role in the evolution of CRC pathologic stage for the first time by graph-based clustering. Besides, the 3 hub genes were later identified (HOXC6, HOXC8 and BMP5) via pseudotime, correlation and survival analysis. Importantly, instead of Multi-Omics Matrix Factorization 34, a more stable and effective algorithm CIBERSORT was performed to construct the cell infiltration classifier, which divided cells in bulk tumor tissue into 2 classifications (C1: HOXC6 ^high^, HOXC8^ high^, BMP5 ^low^; C2: HOXC6 ^low^, HOXC8 ^low^, BMP5^ high^). Independent GEO datasets strongly validated that patients in C2 group with upregulated BMP5 and downregulated HOXC6/8 were related to lower pathologic stage and better clinical survival. Our study demonstrated that HOXC6, HOXC8 as well as BMP5 were implicated in the pathologic evolution and a classifier on the basis of the expression of the 3 genes could serve as a prognostic factor, which would facilitate the clinical survival forecast for CRC patients.

Notably, the 3 hub genes were all involved in TGF-β signaling pathway 35-38. Recent researches emphasized that TGF-β signaling inhibited proliferation and proceeded apoptosis in CRC epithelial cells 39-41. Escaping the growth-inhibiting effect of TGF-β signaling in tumor epithelial cells promoted CRC development 7. This signaling was also involved in epithelial-mesenchymal transition (EMT) to induce CRC metastasis 42 as well as T cell exclusion and immune failure in tumor immune microenvironment 43. A Pan-Cancer Analysis highlighted that gene alteration in TGF-β pathway was carried by 39% cancers, especially gastrointestinal (GI) cancer, and BMP5 was one of the 6 recurrent hotspot mutations in GI cancers 44. In fact, BMP5 was identified as a tumor suppressor in sporadic CRC and the loss of BMP5 happening at early stages of CRC was linked to the poor survival of patients 45. In addition, Romagnoli M revealed that BMP5 was repressed by Blimp-1 during EMT process via TGF-β1 in breast cancer and that the poor prognosis of breast cancer was associated with BMP5 low expression 46. HOXC6 was overexpressed in multiple solid tumors, like hepatocellular carcinoma, cervical carcinoma (CC), head and neck cancer and GI carcinoids 47-50. In CC, HOXC6 silencing repressed the activation of TGF-β signaling pathway via blocking smad-4 phosphorylation, thus inhibiting cell proliferation and EMT in CC cells 51. A human tissue microarray containing 462 samples from CRC patients detected significantly higher HOXC6 expression in tumor tissues compared to matched normal mucosa (P<0.001), which also acted as an independent prognostic marker to poor overall survival 52. Moreover, HOXC6 deregulation decreased CRC cell growth mediated by the suppression of autophagy directly or indirectly 52. HOXC8, in the same homeobox family with HOXC6, was reported to serve as a transcription activator to boost the expression of TGFβ1, leading to an increase of the proliferation, anchorage-independent growth and migration in NSCLC 53. In CRC, BMP signaling functioned as a crucially inhibitory element in tumorigenesis 54, where HOXC8 was discovered to be a negative regulator together with smad6 55. Moreover, GSEA results of high and low C1/C2 ratio groups implied the contribution of TGF-β signaling pathway in clinical prognosis of CRC patients. Hence, it made sense to elucidate that the 3 genes were recruited in our cell infiltration classifier and performed well in pathologic stage and prognosis forecast. In terms of the differential expression in epithelial and T cells, it was revealed that highly transcriptional BMP family signatures including BMP5 promoted T cell infiltration in estrogen receptor-positive breast cancer 56. However, the effect and mechanism of BMP5 expressed in T cells remained unclear.

There were still some limitations in our current study that should be considered when elucidating the results of our findings. First, an essential step might be verification with clinical single-cell data, taking into account of the distinction between bulk sequencing and single-cell technique. In addition, although the function of HOXC6 has been verified in clinical CRC patients 52, further experiments *in vivo* and *in vitro* are still required. More explorations aimed at the crosstalk of the 3 hub genes might shed light on the underlying mechanism.

Collectively, technology advance toward single-cell sequencing enhances our recognition of tumor heterogeneity. The excavation of pathologic stage-related genes facilitates the discovery of novel therapeutic targets. Our study provided a CRC classifier of pathologic stage and survival with potential clinical significance.

## Conclusion

In this study, we identified pathologic evolution related genes in scRNA-Seq and proposed a novel specific cell infiltration classifier to prognosis prediction of CRC patients. Patients with upregulated BMP5 and downregulated HOXC6 and HOXC8 were related to lower pathologic stages and better prognosis. These hub genes also suggested the potentially crucial role of TGF-β signaling pathway in CRC tumorigenesis and progression.

## Supplementary Material

Supplementary figures and tables.

## Figures and Tables

**Table 1:** General property information of validation datasets

Dataset	TCGA (COAD&READ)	GSE39582	GSE37892	GSE12945	GSE17537	GSE17538
Sample capacity	363	552	130	62	55	232
Data type	RNA-Seq	Gene chip	Gene chip	Gene chip	Gene chip	Gene chip
Standardization method	TPM	RMA	RMA	RMA	RMA	RMA
Clinical characteristic	overall survival	overall survival	pathologic stage	overall survival	overall survival& pathologic stage	overall survival& pathologic stage
Median age (IQR)	56.57-76.29	59.00- 76.00	59.25-76.00	59.00-73.75	54.00- 72.00	56.00- 74.00
Gender ratio (M/F)	1.18	1.22	1.13	1.21	0.90	1.11
Survival (Median year)	6.94	12.08	NA	not arrive median survival	not arrive median survival	11.24

**Table 2:** Results of prognosis analyses with the 3 hub genes in TCGA colorectal cancer

Gene symbol	*P*-value (KM)	HR (KM)	*P*-value (COX)	HR (COX)
HOXC6	3.00E-03	1.90	3.12E-04	1.16
HOXC8	2.90E-03	1.90	2.92E-04	1.23
BMP5	1.20E-02	0.57	3.70E-04	0.83

**Table 3:** Results of correlation analysis between pseudotime and expression levels of hub genes

Gene symbol	Cell population	*P* value	R
HOXC6	epithelial cells	1.95E-02	-0.14
HOXC8	epithelial cells	7.50E-03	-0.16
BMP5	epithelial cells	1.33E-05	0.26
BMP5	T cells	4.10E-03	0.48

## Abbreviations

CRC: colorectal cancer
CMS: consensus molecular subtypes
TGF-β: transforming growth factor-β
scRNA-Seq: single-cell RNA sequencing
GEO: gene expression omnibus
TCGA: the cancer genome atlas
COAD: colonic adenocarcinoma
READ: rectum adenocarcinoma
RNA-Seq: RNA sequencing
TPM: Transcripts Per Million
RMA: robust multi-array average
PCA: principal components analysis
t-SNE: t-distributed stochastic neighbor embedding
SNN: shared nearest neighbor
GSEA: gene set enrichment analysis
CIBERSORT: cell-type identification by estimating relative subsets of RNA Transcripts
EMT: epithelial-mesenchymal transition
GI: gastrointestinal
CC: cervical carcinoma
