# Supplementary material for: Pathologic evolution-related Gene Analysis based on both single-cell and bulk transcriptomics in Colorectal Cancer
Source: J Cancer. 2020 Oct 22;11(23):6861–73. doi: 10.7150/jca.49262 (PMC7591993; doi:10.7150/jca.49262)
Supplement: Supplementary file 1 — Supplementary figures and tables. [file jcav11p6861s1.pdf]

Supplementary Material

Figure S1 Results of t-SNE dimensional reduction in GSE81861

A) Colorectal tumor tissue and normal mucosa were separated distinctly by t-SNE algorithm. B) Different cell populations were separated distinctly by t-SNE algorithm.

A

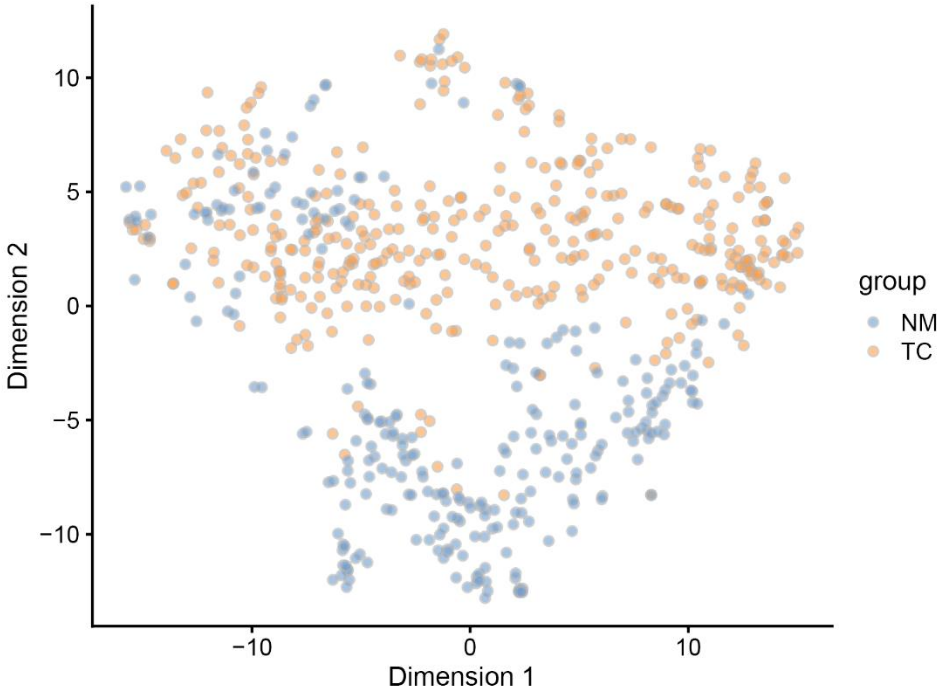

B

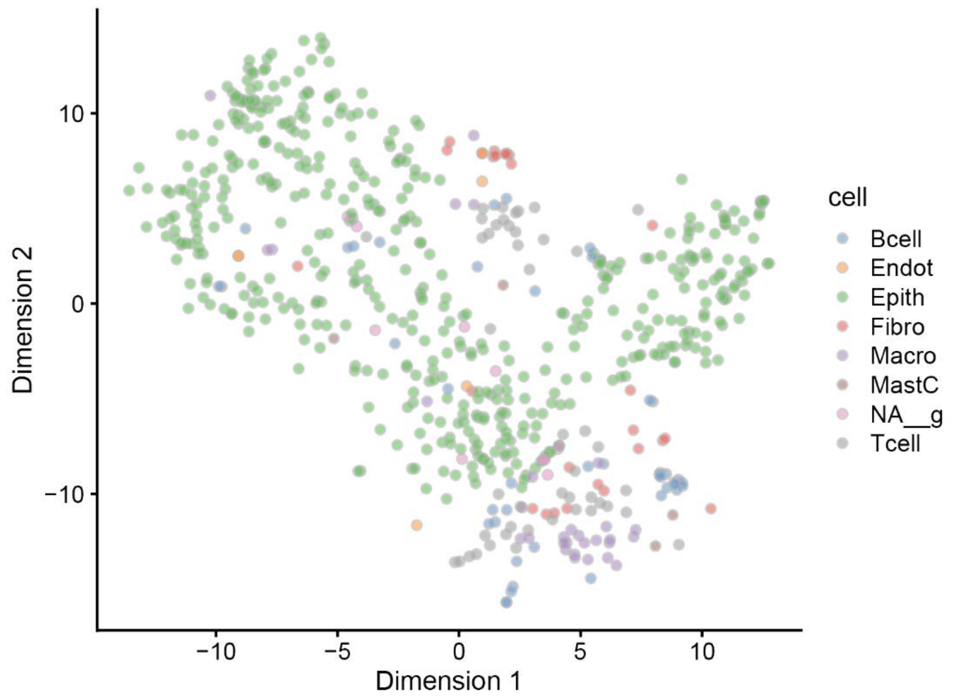

Figure S2 Hub gene expressions respectively in different cell populations.

A) BMP5 was activated in both tumor epithelial cells and T cells. The mean expression in 2 cell populations were displayed in histogram. B) The cell population-expression scatter diagrams of BMP5. BMP5 were expressed in both tumor epithelial and T cells. C) The cell population-expression scatter diagrams of HOXC6. HOXC6 were expressed only in epithelial cells. D) The cell population-expression scatter diagrams of HOXC8. HOXC8 were expressed only in epithelial cells.

A

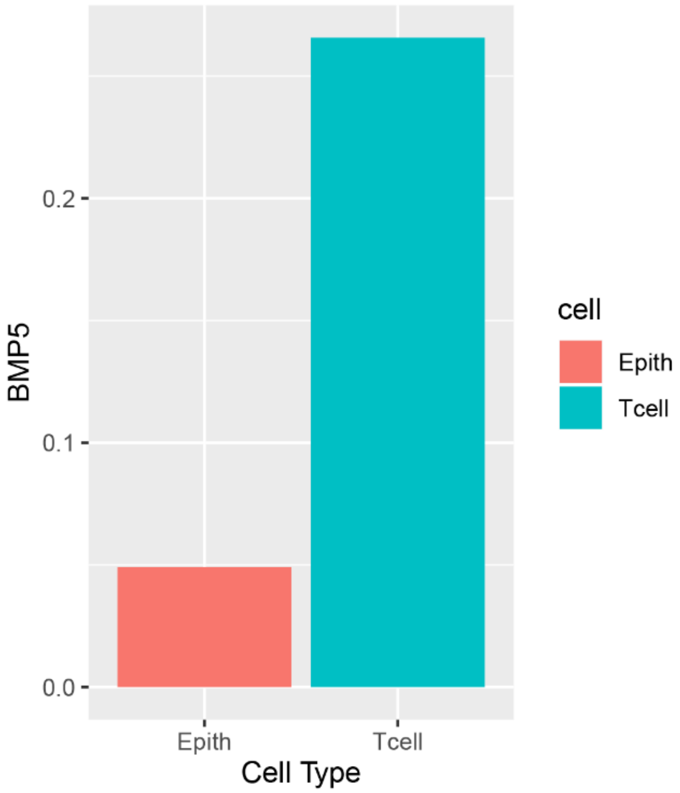
0.2
0.1
0.0
BMP5
Epith
Tcell
Cell Type
cell
Epith
Tcell

B

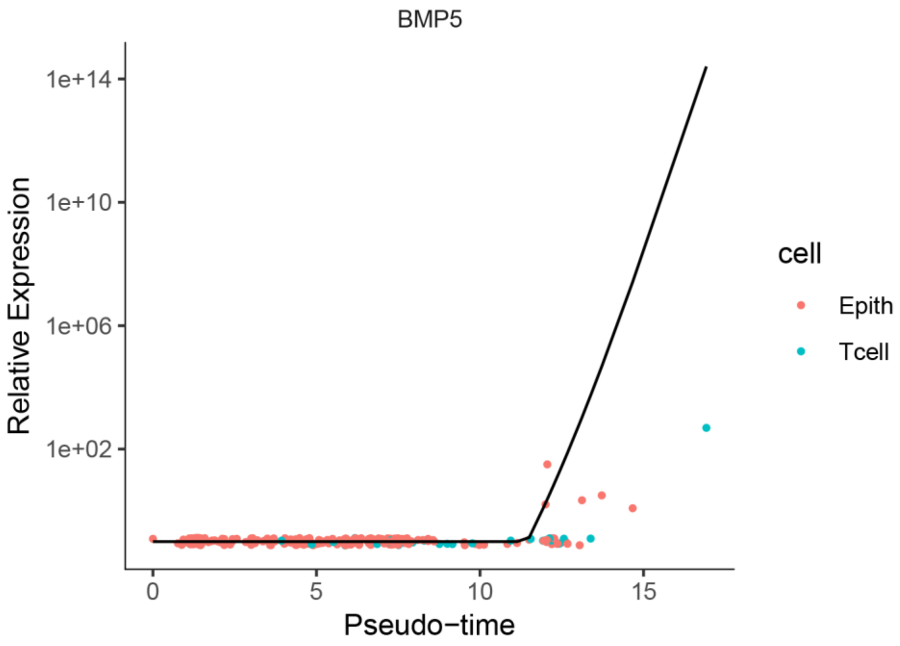
BMP5
1e+14
1e+10
1e+06
1e+02
Relative Expression
0
5
10
15
Pseudo−time
cell
Epith
Tcell

C

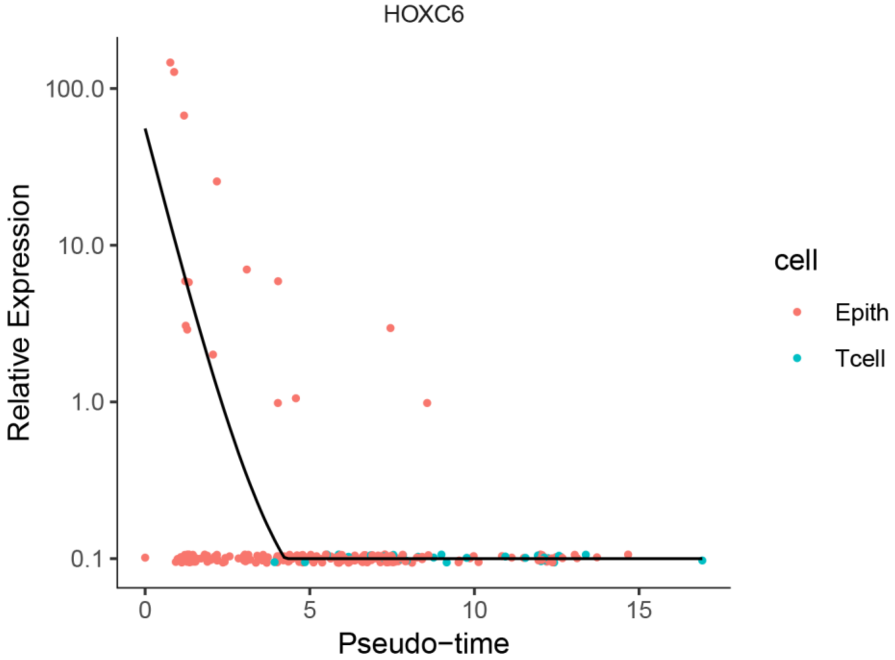
HOXC6
100.0
10.0
1.0
0.1
Relative Expression
0
5
10
15
Pseudo−time
cell
Epith
Tcell

D

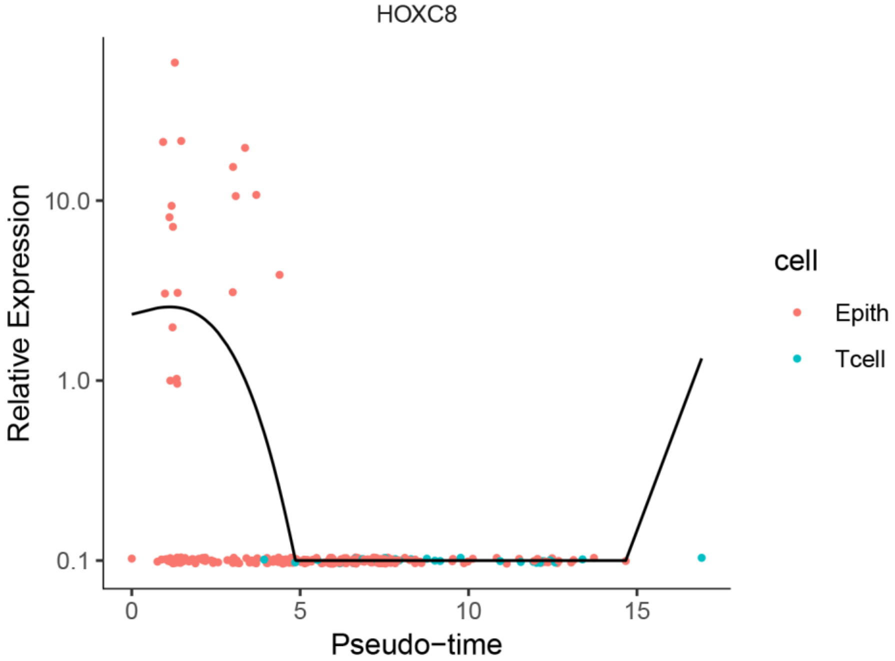
HOXC8
10.0
1.0
0.1
Relative Expression
0
5
10
15
Pseudo−time
cell
Epith
Tcell

Table S1: Cluster of Epithelial cells

cluster	Number of high pathologic stage	Number of low pathologic stage
1	0	26
2	47	0
3	35	17
4	11	53
5	1	17
6	4	61

Table S2: Cluster of T cells

cluster	Number of high pathologic stage	Number of low pathologic stage
1	24	0
2	0	10

Table S3: Significant upregulated marker genes in low pathologic-stage-related cluster of epithelial cells

Gene Symbol	cluster	P value	logFC.1	logFC.2	logFC.3	logFC.4	logFC.5	logFC.6
KLK7	1	3.10E-07	0	4.5596	4.4434	4.5358	4.0112	4.4560
TNFRSF11B	1	4.10E-07	0	6.1323	6.3014	6.2171	5.4781	6.3014
AHNAK2	1	5.59E-07	0	4.7181	4.6769	4.4947	3.2358	4.5125
KLK6	1	1.61E-05	0	3.8731	3.4256	3.5945	3.4884	3.0954
DHRS9	1	4.37E-04	0	3.2437	3.3554	3.1990	3.2345	3.3554
RP11-635N19.3	1	1.10E-03	0	2.7666	2.6982	2.6112	1.1721	-0.3589
IRX2	1	3.51E-03	0	1.8754	1.8340	1.7835	1.9227	1.9227
ABLIM3	1	3.84E-03	0	1.2070	1.3051	1.2814	1.0009	0.8797
SDCBP2	1	5.62E-03	0	2.1064	2.7380	2.3849	1.1817	2.1033
RRAS	1	7.10E-03	0	1.8187	1.8324	1.8007	1.2438	1.0240
LYPD3	1	7.52E-03	0	1.6993	1.6993	1.5192	1.4917	1.6993
CCL5	1	8.34E-03	0	-0.2546	1.2678	1.0646	0.1558	1.0502
CD99P1	1	8.73E-03	0	1.4944	1.5748	1.6073	1.0444	0.8875
CLDN10-AS1	1	1.07E-02	0	1.5522	1.5038	1.5522	1.4876	1.5522
MMP10	1	1.15E-02	0	1.8291	1.8291	1.7861	1.6237	1.8291
EPB41L3	1	1.18E-02	0	1.7195	1.8874	1.6999	1.6374	1.8874
ABCA10	1	1.24E-02	0	1.6050	1.4326	1.6050	1.3519	1.6050
CDKN2B	1	1.51E-02	0	1.6138	1.6111	1.5330	1.0337	1.5817
MMP1	1	1.88E-02	0	1.8642	1.8642	1.8061	1.5316	1.8642
FGF2	1	1.98E-02	0	1.6315	1.6844	1.6844	1.6844	1.6844
FLRT3	1	2.05E-02	0	1.1629	1.4617	1.4617	1.3332	1.2178
SULT1E1	1	2.18E-02	0	1.5470	1.5470	1.5470	1.2997	1.3818
HSPA7	1	2.22E-02	0	1.4945	1.3155	1.4975	0.8721	1.2497
LINC00704	1	2.99E-02	0	1.2223	1.3389	1.2744	1.0568	1.3389
TPTE	1	3.27E-02	0	0.1656	0.4954	0.4867	0.4464	0.4954
TFPI	1	3.48E-02	0	2.1048	1.8221	1.6660	2.0336	0.9363
PCNXL2	1	3.77E-02	0	1.3738	1.8768	2.1729	1.3866	1.6438
MGP	1	3.87E-02	0	1.0767	1.1606	1.1518	1.1170	1.1606
RP11-884K10.7	1	4.08E-02	0	1.5819	1.9270	1.7554	1.1640	0.3673
KRT6C	1	4.47E-02	0	0.5137	0.5137	0.5137	0.4781	0.5137
ZNF736P9Y	1	4.91E-02	0	1.2327	1.2327	1.2327	1.2327	1.1033
SDAD1P1	1	4.93E-02	0	1.1276	1.2263	1.2263	1.0235	1.2263
AHNAK2	5	4.01E-03	-3.2358	1.4823	1.4411	1.2589	0.0000	1.2767
RP11-635N19.3	5	4.86E-03	-1.1721	1.5944	1.5261	1.4391	0.0000	-1.5310
CDHR2	5	1.91E-02	-0.4254	0.8511	1.0790	0.9546	0.0000	0.9580
CCL5	5	2.04E-02	-0.1558	-0.4104	1.1120	0.9088	0.0000	0.8944
BCL2L13	5	2.24E-02	1.13892	1.7151	1.4688	1.7426	0.0000	0.4675
TMEM74B	5	2.38E-02	0.55255	0.8105	0.8105	0.8041	0.0000	-0.0950
LA16c-358B7.3	5	2.72E-02	0.88808	0.8104	0.9234	0.4711	0.0000	0.3039

TNFRSF11B	5	3.10E-02	-5.4781	0.6542	0.8233	0.7390	0.0000	0.8233
RP11-401P9.4	5	4.53E-02	0.46354	0.7512	0.8053	0.7386	0.0000	0.6505
LEMD1	5	4.90E-02	-0.2756	0.7289	0.7289	0.7226	0.0000	-0.0306
LEF1	6	1.86E-04	2.3873	2.2546	2.3873	2.1026	2.1609	0.0000
RP11-635N19.3	6	2.18E-04	0.3589	3.1255	3.0571	2.9701	1.5310	0.0000
EIF4BP6	6	2.44E-04	1.87981	1.8006	1.1534	1.8438	1.4307	0.0000
MAATS1	6	4.73E-04	1.92809	1.8782	2.0028	1.9574	1.8681	0.0000
TMEM191A	6	5.75E-04	1.50267	1.5848	1.5318	1.5009	1.2328	0.0000
GRB14	6	9.34E-04	2.77527	2.7753	2.7753	2.6206	2.5145	0.0000
AC108938.2	6	1.70E-03	1.3654	1.2686	1.2302	1.2194	1.1902	0.0000
DNM1	6	2.11E-03	1.22623	1.6911	1.7896	1.7206	1.1273	0.0000
GRAMD1C	6	2.34E-03	1.95532	1.9738	2.1866	2.0711	2.1650	0.0000
RP11-104J23.1	6	3.85E-03	0.44466	0.4214	0.4447	0.2323	0.3126	0.0000
ALPPL2	6	7.70E-03	1.30457	1.6016	1.3586	1.4869	1.1948	0.0000
WNT3	6	7.76E-03	1.42722	2.0813	2.0115	1.9928	1.7051	0.0000
MTBP	6	8.02E-03	1.62377	1.6244	1.3139	1.5394	1.0746	0.0000
HOXC8	6	8.15E-03	0.53415	1.0375	1.0375	0.9123	0.9330	0.0000
AC023490.1	6	9.78E-03	0.59026	0.6251	0.6251	0.5932	0.5861	0.0000
ANKEF1	6	9.87E-03	1.08362	1.2917	1.1868	1.1793	1.0161	0.0000
OTX1	6	1.06E-02	1.23123	1.1397	1.2743	1.2108	1.0622	0.0000
ADPRHL2	6	1.07E-02	1.73038	1.9153	1.5416	1.8006	1.7661	0.0000
AQP1	6	1.09E-02	1.24017	1.5805	1.6243	1.4998	1.1784	0.0000
AC013268.2	6	1.40E-02	0.81656	0.8166	0.6835	0.7812	0.8166	0.0000
S100A9	6	1.41E-02	1.08399	0.8748	1.1609	1.0753	0.8764	0.0000
RP11-480A16.1	6	1.69E-02	1.3315	1.3123	1.5085	1.3831	1.1860	0.0000
EPDR1	6	1.74E-02	0.79968	1.5337	1.5337	1.2164	1.3921	0.0000
LRTOMT	6	1.91E-02	1.3206	1.2977	1.3924	1.2935	0.7854	0.0000
TMEM74B	6	2.15E-02	0.64752	0.9055	0.9055	0.8991	0.0950	0.0000
PLA2G4A	6	2.40E-02	1.43172	1.4449	1.7436	1.7348	1.3457	0.0000
DGAT2	6	2.69E-02	1.18659	1.1650	1.2999	1.2131	1.1567	0.0000
FGF20	6	2.72E-02	0.04874	0.9949	0.9949	0.9211	0.6782	0.0000
PROS1	6	2.73E-02	1.27796	1.1892	0.8135	1.1124	0.9712	0.0000
C5orf54	6	2.74E-02	1.07506	1.0340	1.0751	1.0350	0.9613	0.0000
RP11-30P6.6	6	2.92E-02	1.34383	1.1759	1.1286	1.2335	0.7522	0.0000
RNU1-106P	6	2.96E-02	0.34236	0.5291	0.5291	0.4906	0.4653	0.0000
RBP2	6	3.06E-02	1.17901	1.1514	1.1286	1.1113	1.0858	0.0000
TRAV30	6	3.27E-02	0.46491	0.7788	0.7788	0.7788	0.7034	0.0000
BOK-AS1	6	3.57E-02	0.55163	0.5516	0.5516	0.4038	0.5387	0.0000
RP11-197P3.5	6	3.59E-02	0.86243	0.7962	0.8487	0.8624	0.8033	0.0000
LRRC20	6	3.65E-02	0.94931	0.9383	0.8773	0.8502	0.7570	0.0000
CTC-365E16.1	6	3.68E-02	1.39991	1.4176	1.3571	1.4610	1.4610	0.0000
IGIP	6	3.74E-02	0.58169	0.9534	1.0354	1.0402	0.7282	0.0000
POU5F2	6	3.93E-02	1.05257	1.0782	1.1414	0.9757	0.6950	0.0000

PREX1	6	3.94E-02	0.84269	0.6778	0.5614	0.7815	0.7904	0.0000
TRIM32	6	4.22E-02	1.32588	1.4416	1.4248	1.5260	1.4072	0.0000
C3orf14	6	4.24E-02	0.47399	1.0847	1.1868	1.2763	1.0542	0.0000
RP11-181C3.1	6	4.47E-02	0.81825	0.8119	0.8183	0.7746	0.7742	0.0000
SEMG1	6	4.79E-02	0.63376	0.7831	0.9081	0.8832	0.8733	0.0000
RP11-271C24.3	6	4.91E-02	0.69059	0.9988	1.0187	1.0999	0.8327	0.0000

Note: logFC.x is log2(Fold change) of cluster x vs. gene high expression cluster

Table S4: Significant upregulated marker genes in low pathologic-stage-related cluster of T cells

Gene symbol	Cluster	P value	logFC.1
YDJC	2	0.1385	0.8333
MBOAT7	2	0.1467	0.6635
SERINC4	2	0.1473	0.6233
SH3YL1	2	0.1475	0.6521
IMPACT	2	0.1481	0.6717
CHAF1A	2	0.1493	0.5733
TNFSF13B	2	0.1496	0.7236
UVSSA	2	0.1497	0.7272
JKAMP	2	0.1500	0.7394
PPIL3	2	0.1512	0.8403
MSS51	2	0.1516	0.8610
TMED7	2	0.1520	0.6689
PAXBP1-AS1	2	0.1541	0.7658
SEC22A	2	0.1541	0.7669
ECT2	2	0.1544	0.6246
SLC17A4	2	0.1544	0.6274
TNFSF8	2	0.1546	0.7956
AC145110.1	2	0.1557	0.8610
TRIM36-IT1	2	0.1685	0.8987
FCRL6	2	0.1685	0.8948
CDYL2	2	0.1685	0.8947
LARP1B	2	0.1685	0.8889
ZBED5-AS1	2	0.1685	0.8726
KLHL12	2	0.1685	0.8725
L2HGDH	2	0.1685	0.8655
BTLA	2	0.1685	0.8622
CCR8	2	0.1685	0.7631
CD27	2	0.1685	0.7477
CDK4	2	0.1685	0.5870
CDC34	2	0.1685	0.4972

Note: logFC.x is log2(Fold change) of cluster x vs. gene high expression cluster

Table S5：Results of spearman correlation analysis with the 84 pathologic-stage-related genes

Gene Symbol	P value	Spearman R
DOCK10	2.49E-10	0.352
DNAH9	3.63E-09	0.329
HOPX	1.03E-07	0.298
PRUNE2	1.15E-07	0.297
FCER1G	1.34E-07	0.296
TOX	5.07E-07	0.282
NKG7	9.49E-07	0.276
SYTL3	1.63E-06	0.270
TEX28P2	5.60E-06	0.256
AC096579.7	8.78E-06	0.251
DPF3	9.84E-06	0.250
TTC39DP	1.07E-05	0.249
GNLY	1.79E-05	0.243
AC092535.3	1.97E-05	0.241
PPP1R16B	2.02E-05	0.241
CD40LG	2.25E-05	0.240
KLRC2	2.76E-05	0.237
IL5RA	3.76E-05	0.233
ZNF385D	4.22E-05	0.232
CCDC141	4.26E-05	0.232
BMP5	4.69E-05	0.231
VWA3B	5.33E-05	0.229
TRGC1	5.41E-05	0.229
IFNG	5.62E-05	0.228
RBM20	5.76E-05	0.228
AC008964.1	6.06E-05	0.227
SKIDA1	6.12E-05	0.227
RNF212	6.73E-05	0.226
GPR171	7.34E-05	0.225
TAGAP	7.51E-05	0.224
MDFIC	7.56E-05	0.224
MCAM	8.52E-05	0.223
UBASH3A	9.53E-05	0.221
NEB	0.00010993	0.219
ARHGAP9	0.00012861	0.217
EEF1DP3	0.00012985	0.217
BIN2	0.00013204	0.217
TEX28	0.0001346	0.217

INSL6	0.0001499	0.215
FAM159A	0.00015469	0.215
SLC24A2	0.0001551	0.215
ITGB2	0.00016591	0.214
GVINP1	0.00016618	0.214
CTSW	0.00016717	0.214
IGHA2	0.00021732	0.210
FMNL1	0.00022617	0.209
CD226	0.00022955	0.209
ZBTB16	0.0002374	0.209
COL1A1	0.00023874	0.209
ATP8B4	0.00024202	0.208
DUSP26	0.00025865	0.207
ZNF85	0.0002772	0.206
ABI3	0.00030717	0.205
ADAMTS20	0.00031935	0.204
ZNF418	0.00034596	0.203
AL022393.7	0.00034617	0.203
SLC25A5P4	0.00039604	0.201
IPCEF1	0.0004056	0.201
EMX2OS	0.00041162	0.201
GNGT2	0.00041598	0.201
NCAM1	0.00041709	0.201
EYA2	0.0004173	0.201
CCR5	0.00042151	0.200
ADAMTSL1	0.0004282	0.200
ST3GAL6	0.00041541	-0.201
RN7SL698P	0.00040891	-0.201
MMP10	0.00037953	-0.202
FGF19	0.00036621	-0.202
BICD1	0.00025373	-0.208
LYPD3	0.00018308	-0.212
RPS4XP14	0.00016205	-0.214
ABCA10	0.00011435	-0.219
TRAV30	5.94E-05	-0.227
CST1	5.67E-05	-0.228
NUDT13	5.57E-05	-0.228
HOXC6	5.40E-05	-0.229
AQP1	3.61E-05	-0.234
SULT1E1	3.55E-05	-0.234
WNT3	2.42E-05	-0.239
DPY19L1P2	9.71E-06	-0.250
TMEM74B	1.15E-06	-0.274

LEMD1	1.18E-07	-0.297
FGF20	3.80E-08	-0.308
HOXC8	1.72E-08	-0.315

Table S6: gene expression signatures reference for different cell categories

Gene symbol	C1 Cells	C2 Cells
HOXC6	High quartile	Low quartile
HOXC8	High quartile	Low quartile
BMP5	Low quartile	High quartile

Table S7: Results of CIBERSORT with CRC patients in TCGA

Mixture	C1 Cells	C2 Cells	Correlation	RMSE
TCGA_3L_AA1B	0.00	1.00	0.94	0.91
TCGA_4N_A93T	0.00	1.00	0.86	0.92
TCGA_4T_AA8H	0.00	1.00	0.43	0.94
TCGA_5M_AAT4	0.85	0.15	0.97	0.43
TCGA_5M_AAT5	0.85	0.15	0.97	0.43
TCGA_5M_AAT6	1.00	0.00	1.00	0.57
TCGA_5M_AATA	0.00	1.00	0.45	0.94
TCGA_5M_AATE	0.00	1.00	0.43	0.94
TCGA_A6_2672	0.86	0.14	1.00	0.39
TCGA_A6_2674	1.00	0.00	0.82	0.80
TCGA_A6_2675	0.00	1.00	0.51	0.94
TCGA_A6_2677	0.00	1.00	0.80	0.92
TCGA_A6_2682	0.86	0.14	0.97	0.42
TCGA_A6_2684	0.86	0.14	1.00	0.39
TCGA_A6_2685	0.00	1.00	0.77	0.92
TCGA_A6_2686	0.85	0.15	0.97	0.43
TCGA_A6_3809	0.57	0.43	0.46	0.77
TCGA_A6_3810	0.00	1.00	0.56	0.93
TCGA_A6_4105	0.00	1.00	0.99	0.91
TCGA_A6_5656	0.84	0.16	0.91	0.51
TCGA_A6_5657	0.86	0.14	0.98	0.42
TCGA_A6_5659	0.00	1.00	0.88	0.92
TCGA_A6_5660	0.00	1.00	0.78	0.92
TCGA_A6_5661	0.86	0.14	0.98	0.42
TCGA_A6_5662	0.61	0.39	0.49	0.77
TCGA_A6_5664	0.00	1.00	0.93	0.92

TCGA_A6_5665	0.86	0.14	0.98	0.42
TCGA_A6_5666	0.86	0.14	0.98	0.41
TCGA_A6_5667	0.00	1.00	0.48	0.94
TCGA_A6_6137	0.00	1.00	0.52	0.94
TCGA_A6_6138	0.00	1.00	0.54	0.93
TCGA_A6_6140	0.00	1.00	0.40	0.94
TCGA_A6_6141	0.86	0.14	0.98	0.41
TCGA_A6_6142	0.86	0.14	0.99	0.40
TCGA_A6_6648	0.00	1.00	0.45	0.94
TCGA_A6_6649	0.85	0.15	0.95	0.45
TCGA_A6_6650	0.00	1.00	0.46	0.94
TCGA_A6_6651	0.73	0.27	0.63	0.73
TCGA_A6_6652	0.00	1.00	0.44	0.94
TCGA_A6_6653	0.85	0.15	0.96	0.44
TCGA_A6_6654	0.84	0.16	0.93	0.48
TCGA_A6_6780	1.00	0.00	1.00	0.57
TCGA_A6_6781	0.86	0.14	1.00	0.39
TCGA_A6_6782	0.84	0.16	0.92	0.50
TCGA_A6_A565	0.00	1.00	0.44	0.94
TCGA_A6_A566	0.85	0.15	0.96	0.44
TCGA_A6_A567	0.00	1.00	0.38	0.94
TCGA_A6_A56B	0.00	1.00	0.43	0.94
TCGA_A6_A5ZU	0.86	0.14	1.00	0.39
TCGA_AA_3489	1.00	0.00	1.00	0.57
TCGA_AA_3492	0.85	0.15	0.96	0.44
TCGA_AA_3495	0.00	1.00	0.58	0.93
TCGA_AA_3496	0.48	0.52	0.42	0.76
TCGA_AA_3502	0.00	1.00	0.44	0.94
TCGA_AA_3506	0.39	0.61	0.40	0.76
TCGA_AA_3511	0.00	1.00	0.52	0.93
TCGA_AA_3526	0.00	1.00	0.91	0.92
TCGA_AA_3655	0.00	1.00	0.45	0.94
TCGA_AA_3660	0.00	1.00	0.49	0.94
TCGA_AA_3663	0.85	0.15	0.96	0.44
TCGA_AA_3675	0.00	1.00	0.96	0.91
TCGA_AA_3685	0.00	1.00	0.40	0.94
TCGA_AA_3712	0.00	1.00	0.59	0.93
TCGA_AA_3713	0.85	0.15	0.96	0.44
TCGA_AA_A01P	0.86	0.14	0.98	0.42
TCGA_AA_A01X	0.00	1.00	0.80	0.92
TCGA_AA_A01Z	0.82	0.18	0.85	0.57
TCGA_AA_A02Y	0.00	1.00	0.52	0.94
TCGA_AD_5900	0.85	0.15	0.97	0.43

TCGA_AD_6548	0.00	1.00	0.43	0.94
TCGA_AD_6888	0.00	1.00	0.59	0.93
TCGA_AD_6889	0.85	0.15	0.96	0.44
TCGA_AD_6890	0.81	0.19	0.81	0.61
TCGA_AD_6895	0.85	0.15	0.97	0.43
TCGA_AD_6899	0.71	0.29	0.60	0.74
TCGA_AD_6901	0.86	0.14	0.98	0.41
TCGA_AD_6963	0.00	1.00	0.57	0.93
TCGA_AD_6964	0.86	0.14	0.97	0.42
TCGA_AD_6965	0.80	0.20	0.78	0.64
TCGA_AD_A5EJ	0.85	0.15	0.97	0.43
TCGA_AD_A5EK	0.00	1.00	0.54	0.93
TCGA_AF_2687	0.82	0.18	0.83	0.59
TCGA_AF_2690	0.70	0.30	0.58	0.75
TCGA_AF_2693	0.00	1.00	0.43	0.94
TCGA_AF_3911	0.00	1.00	0.43	0.94
TCGA_AF_4110	0.00	1.00	0.43	0.94
TCGA_AF_5654	0.00	1.00	0.59	0.93
TCGA_AF_6136	0.00	1.00	0.45	0.94
TCGA_AF_6655	0.00	1.00	0.48	0.94
TCGA_AF_6672	0.00	1.00	0.43	0.94
TCGA_AF_A56K	0.00	1.00	0.49	0.94
TCGA_AF_A56L	0.00	1.00	0.93	0.92
TCGA_AF_A56N	0.00	1.00	0.49	0.94
TCGA_AG_3591	0.00	1.00	0.43	0.94
TCGA_AG_3592	0.00	1.00	0.40	0.94
TCGA_AG_3725	0.00	1.00	0.42	0.94
TCGA_AG_3731	0.00	1.00	0.57	0.93
TCGA_AG_3732	0.00	1.00	0.46	0.94
TCGA_AG_3742	0.00	1.00	0.48	0.94
TCGA_AG_4021	0.85	0.15	0.96	0.43
TCGA_AG_4022	0.00	1.00	0.44	0.94
TCGA_AH_6544	0.00	1.00	0.46	0.94
TCGA_AH_6547	0.00	1.00	0.49	0.94
TCGA_AH_6643	1.00	0.00	1.00	0.57
TCGA_AH_6644	0.00	1.00	0.48	0.94
TCGA_AH_6897	0.00	1.00	0.49	0.94
TCGA_AH_6903	0.00	1.00	0.44	0.94
TCGA_AM_5820	0.85	0.15	0.96	0.45
TCGA_AM_5821	0.00	1.00	0.48	0.94
TCGA_AU_3779	0.81	0.19	0.82	0.60
TCGA_AU_6004	0.83	0.17	0.89	0.53
TCGA_AY_5543	0.00	1.00	0.70	0.93

TCGA_AY_6196	0.86	0.14	1.00	0.39
TCGA_AY_6197	0.00	1.00	0.99	0.91
TCGA_AY_6386	0.00	1.00	0.54	0.93
TCGA_AY_A54L	0.00	1.00	0.84	0.92
TCGA_AY_A69D	0.00	1.00	0.46	0.94
TCGA_AY_A71X	0.00	1.00	0.53	0.93
TCGA_AY_A8YK	0.00	1.00	0.49	0.94
TCGA_AZ_4313	0.85	0.15	0.96	0.44
TCGA_AZ_4315	0.82	0.18	0.86	0.56
TCGA_AZ_4323	0.85	0.15	0.97	0.43
TCGA_AZ_4614	0.86	0.14	0.99	0.39
TCGA_AZ_4615	0.85	0.15	0.97	0.43
TCGA_AZ_4616	0.86	0.14	0.98	0.42
TCGA_AZ_4682	0.00	1.00	0.45	0.94
TCGA_AZ_4684	0.84	0.16	0.92	0.50
TCGA_AZ_5403	0.00	1.00	0.46	0.94
TCGA_AZ_5407	0.00	1.00	0.45	0.94
TCGA_AZ_6598	0.85	0.15	0.97	0.43
TCGA_AZ_6599	0.85	0.15	0.96	0.44
TCGA_AZ_6600	0.84	0.16	0.92	0.50
TCGA_AZ_6603	0.00	1.00	0.54	0.93
TCGA_AZ_6605	0.77	0.23	0.71	0.68
TCGA_AZ_6606	0.83	0.17	0.88	0.54
TCGA_AZ_6607	0.86	0.14	1.00	0.39
TCGA_CA_5254	1.00	0.00	1.00	0.57
TCGA_CA_5255	0.83	0.17	0.89	0.53
TCGA_CA_5256	0.00	1.00	0.44	0.94
TCGA_CA_5796	0.00	1.00	0.43	0.94
TCGA_CA_5797	0.00	1.00	0.42	0.94
TCGA_CA_6715	0.00	1.00	0.67	0.93
TCGA_CA_6716	0.00	1.00	0.44	0.94
TCGA_CA_6717	0.85	0.15	0.95	0.46
TCGA_CA_6718	0.85	0.15	0.97	0.43
TCGA_CA_6719	0.82	0.18	0.85	0.58
TCGA_CI_6619	0.82	0.18	0.86	0.56
TCGA_CI_6620	0.00	1.00	0.75	0.92
TCGA_CI_6621	0.00	1.00	0.45	0.94
TCGA_CI_6622	0.00	1.00	0.94	0.91
TCGA_CI_6623	0.00	1.00	0.43	0.94
TCGA_CI_6624	0.00	1.00	0.44	0.94
TCGA_CK_4947	0.00	1.00	0.53	0.93
TCGA_CK_4948	0.00	1.00	0.77	0.92
TCGA_CK_4950	0.75	0.25	0.65	0.72

TCGA_CK_4951	0.86	0.14	0.99	0.40
TCGA_CK_4952	0.85	0.15	0.96	0.44
TCGA_CK_5912	0.00	1.00	0.58	0.93
TCGA_CK_5913	0.86	0.14	0.99	0.40
TCGA_CK_5914	0.00	1.00	0.43	0.94
TCGA_CK_5915	0.00	1.00	0.43	0.94
TCGA_CK_5916	0.85	0.15	0.97	0.43
TCGA_CK_6746	1.00	0.00	1.00	0.57
TCGA_CK_6747	0.00	1.00	0.46	0.94
TCGA_CK_6748	0.00	1.00	0.82	0.92
TCGA_CK_6751	0.85	0.15	0.96	0.45
TCGA_CL_4957	0.00	1.00	0.97	0.91
TCGA_CL_5917	0.00	1.00	0.45	0.94
TCGA_CL_5918	0.00	1.00	0.46	0.94
TCGA_CM_4743	0.85	0.15	0.97	0.43
TCGA_CM_4744	0.00	1.00	0.53	0.93
TCGA_CM_4747	0.86	0.14	0.98	0.41
TCGA_CM_4751	0.00	1.00	0.88	0.92
TCGA_CM_5344	0.86	0.14	0.98	0.41
TCGA_CM_5348	0.86	0.14	0.99	0.40
TCGA_CM_5349	0.86	0.14	0.98	0.41
TCGA_CM_5860	0.00	1.00	0.94	0.91
TCGA_CM_5861	0.85	0.15	0.97	0.43
TCGA_CM_5862	0.83	0.17	0.88	0.54
TCGA_CM_5863	0.85	0.15	0.95	0.46
TCGA_CM_5864	0.00	1.00	0.64	0.93
TCGA_CM_5868	0.85	0.15	0.94	0.47
TCGA_CM_6161	0.00	1.00	0.43	0.94
TCGA_CM_6162	0.86	0.14	0.98	0.41
TCGA_CM_6163	0.00	1.00	0.97	0.91
TCGA_CM_6164	0.00	1.00	0.82	0.92
TCGA_CM_6165	0.00	1.00	0.56	0.93
TCGA_CM_6166	0.00	1.00	0.92	0.92
TCGA_CM_6167	0.86	0.14	0.99	0.39
TCGA_CM_6168	0.86	0.14	0.98	0.42
TCGA_CM_6169	0.00	1.00	0.69	0.93
TCGA_CM_6170	0.00	1.00	0.62	0.93
TCGA_CM_6171	0.85	0.15	0.96	0.44
TCGA_CM_6172	0.00	1.00	0.47	0.94
TCGA_CM_6674	0.85	0.15	0.97	0.43
TCGA_CM_6675	0.76	0.24	0.69	0.70
TCGA_CM_6676	0.00	1.00	0.91	0.92
TCGA_CM_6677	0.73	0.27	0.62	0.74

TCGA_CM_6678	0.85	0.15	0.96	0.44
TCGA_CM_6679	0.00	1.00	0.54	0.93
TCGA_CM_6680	0.00	1.00	0.65	0.93
TCGA_D5_5537	0.65	0.35	0.52	0.76
TCGA_D5_5538	0.86	0.14	0.98	0.41
TCGA_D5_5539	0.85	0.15	0.96	0.43
TCGA_D5_5540	0.00	1.00	0.94	0.91
TCGA_D5_5541	0.74	0.26	0.65	0.72
TCGA_D5_6529	0.86	0.14	0.98	0.41
TCGA_D5_6530	1.00	0.00	1.00	0.57
TCGA_D5_6531	0.85	0.15	0.97	0.43
TCGA_D5_6532	0.00	1.00	0.50	0.94
TCGA_D5_6533	0.00	1.00	0.44	0.94
TCGA_D5_6534	0.86	0.14	0.99	0.40
TCGA_D5_6535	0.00	1.00	0.92	0.92
TCGA_D5_6536	0.85	0.15	0.97	0.43
TCGA_D5_6537	0.00	1.00	0.85	0.92
TCGA_D5_6538	0.00	1.00	0.99	0.91
TCGA_D5_6539	0.00	1.00	0.43	0.94
TCGA_D5_6540	0.86	0.14	0.97	0.42
TCGA_D5_6541	0.87	0.13	1.00	0.39
TCGA_D5_6898	0.00	1.00	0.43	0.94
TCGA_D5_6920	0.00	1.00	0.43	0.94
TCGA_D5_6922	0.00	1.00	0.51	0.94
TCGA_D5_6923	0.00	1.00	0.60	0.93
TCGA_D5_6924	0.00	1.00	0.62	0.93
TCGA_D5_6926	0.85	0.15	0.96	0.44
TCGA_D5_6927	0.86	0.14	0.99	0.39
TCGA_D5_6928	0.85	0.15	0.97	0.43
TCGA_D5_6929	0.86	0.14	0.99	0.40
TCGA_D5_6930	0.85	0.15	0.96	0.44
TCGA_D5_6931	0.33	0.67	0.40	0.77
TCGA_D5_6932	0.00	1.00	0.54	0.93
TCGA_D5_7000	0.84	0.16	0.90	0.51
TCGA_DC_4745	0.00	1.00	0.43	0.94
TCGA_DC_4749	0.00	1.00	0.43	0.94
TCGA_DC_5337	0.00	1.00	0.46	0.94
TCGA_DC_5869	0.00	1.00	0.48	0.94
TCGA_DC_6154	0.00	1.00	-0.08	0.96
TCGA_DC_6155	0.72	0.28	0.61	0.74
TCGA_DC_6156	0.86	0.14	0.99	0.40
TCGA_DC_6157	0.00	1.00	0.88	0.92
TCGA_DC_6158	0.69	0.31	0.57	0.75

TCGA_DC_6160	0.00	1.00	0.43	0.94
TCGA_DC_6681	0.00	1.00	0.44	0.94
TCGA_DC_6682	0.83	0.17	0.86	0.56
TCGA_DC_6683	0.00	1.00	0.41	0.94
TCGA_DM_A0X9	0.84	0.16	0.93	0.48
TCGA_DM_A0XD	0.86	0.14	0.99	0.40
TCGA_DM_A0XF	0.00	1.00	0.43	0.94
TCGA_DM_A1D0	0.82	0.18	0.85	0.58
TCGA_DM_A1D4	0.00	1.00	0.43	0.94
TCGA_DM_A1D7	0.00	1.00	0.44	0.94
TCGA_DM_A1D8	0.00	1.00	0.81	0.92
TCGA_DM_A1D9	0.00	1.00	0.47	0.94
TCGA_DM_A1DA	0.86	0.14	0.98	0.41
TCGA_DM_A1DB	0.86	0.14	1.00	0.39
TCGA_DM_A1HA	0.86	0.14	0.98	0.42
TCGA_DM_A1HB	0.86	0.14	0.98	0.42
TCGA_DM_A280	1.00	0.00	1.00	0.57
TCGA_DM_A282	0.00	1.00	0.46	0.94
TCGA_DM_A285	0.86	0.14	0.97	0.42
TCGA_DM_A288	0.85	0.15	0.97	0.43
TCGA_DM_A28A	0.86	0.14	0.99	0.40
TCGA_DM_A28C	0.00	1.00	0.42	0.94
TCGA_DM_A28E	0.00	1.00	0.43	0.94
TCGA_DM_A28F	0.00	1.00	0.93	0.92
TCGA_DM_A28G	0.00	1.00	0.46	0.94
TCGA_DM_A28H	0.84	0.16	0.93	0.49
TCGA_DM_A28K	0.80	0.20	0.78	0.64
TCGA_DM_A28M	0.83	0.17	0.89	0.53
TCGA_DT_5265	0.86	0.14	0.97	0.42
TCGA_DY_A0XA	0.87	0.13	1.00	0.39
TCGA_DY_A1DC	0.00	1.00	0.44	0.94
TCGA_DY_A1DD	0.00	1.00	0.56	0.93
TCGA_DY_A1DE	0.69	0.31	0.57	0.75
TCGA_DY_A1DF	0.85	0.15	0.96	0.44
TCGA_DY_A1DG	0.00	1.00	0.39	0.94
TCGA_DY_A1H8	0.00	1.00	0.49	0.94
TCGA_EF_5830	0.00	1.00	0.53	0.93
TCGA_EF_5831	0.00	1.00	0.50	0.94
TCGA_EI_6506	0.00	1.00	0.43	0.94
TCGA_EI_6507	0.85	0.15	0.97	0.43
TCGA_EI_6508	0.00	1.00	0.46	0.94
TCGA_EI_6509	0.85	0.15	0.96	0.44
TCGA_EI_6510	0.00	1.00	0.45	0.94

TCGA_EI_6511	0.00	1.00	0.57	0.93
TCGA_EI_6512	0.00	1.00	0.53	0.93
TCGA_EI_6513	0.59	0.41	0.48	0.77
TCGA_EI_6514	0.00	1.00	0.44	0.94
TCGA_EI_6881	0.00	1.00	0.43	0.94
TCGA_EI_6882	0.00	1.00	0.81	0.92
TCGA_EI_6884	0.00	1.00	0.48	0.94
TCGA_EI_6885	0.00	1.00	0.88	0.92
TCGA_EI_6917	0.86	0.14	0.99	0.40
TCGA_EI_7002	0.00	1.00	0.42	0.94
TCGA_EI_7004	0.00	1.00	0.50	0.94
TCGA_F4_6459	0.77	0.23	0.72	0.68
TCGA_F4_6460	0.00	1.00	0.43	0.94
TCGA_F4_6461	0.85	0.15	0.96	0.44
TCGA_F4_6463	0.84	0.16	0.93	0.48
TCGA_F4_6569	0.86	0.14	0.99	0.41
TCGA_F4_6570	0.85	0.15	0.97	0.43
TCGA_F4_6703	0.85	0.15	0.97	0.43
TCGA_F4_6704	0.00	1.00	0.52	0.94
TCGA_F4_6805	0.00	1.00	0.83	0.92
TCGA_F4_6806	0.00	1.00	0.72	0.93
TCGA_F4_6807	0.85	0.15	0.96	0.45
TCGA_F4_6808	0.00	1.00	0.40	0.94
TCGA_F4_6809	0.86	0.14	1.00	0.39
TCGA_F4_6854	0.00	1.00	0.46	0.94
TCGA_F4_6855	0.85	0.15	0.97	0.43
TCGA_F4_6856	0.84	0.16	0.91	0.51
TCGA_F5_6464	0.85	0.15	0.97	0.43
TCGA_F5_6465	0.00	1.00	0.62	0.93
TCGA_F5_6571	0.00	1.00	0.48	0.94
TCGA_F5_6702	0.83	0.17	0.89	0.53
TCGA_F5_6810	0.00	1.00	0.43	0.94
TCGA_F5_6811	0.61	0.39	0.49	0.77
TCGA_F5_6812	0.83	0.17	0.86	0.56
TCGA_F5_6813	0.78	0.22	0.72	0.68
TCGA_F5_6814	0.86	0.14	0.98	0.42
TCGA_F5_6861	0.00	1.00	0.62	0.93
TCGA_F5_6863	0.00	1.00	0.69	0.93
TCGA_F5_6864	0.86	0.14	0.98	0.41
TCGA_G4_6293	0.00	1.00	0.43	0.94
TCGA_G4_6294	0.00	1.00	0.48	0.94
TCGA_G4_6295	0.86	0.14	0.98	0.41
TCGA_G4_6297	0.25	0.75	0.41	0.79

TCGA_G4_6298	0.85	0.15	0.95	0.46
TCGA_G4_6299	0.85	0.15	0.97	0.43
TCGA_G4_6302	0.85	0.15	0.97	0.43
TCGA_G4_6303	0.00	1.00	0.44	0.94
TCGA_G4_6304	0.85	0.15	0.97	0.43
TCGA_G4_6306	0.00	1.00	0.43	0.94
TCGA_G4_6307	0.00	1.00	0.47	0.94
TCGA_G4_6309	0.00	1.00	0.92	0.92
TCGA_G4_6311	0.86	0.14	0.98	0.42
TCGA_G4_6315	0.00	1.00	0.65	0.93
TCGA_G4_6317	0.00	1.00	0.43	0.94
TCGA_G4_6320	0.85	0.15	0.94	0.47
TCGA_G4_6321	0.00	1.00	0.91	0.92
TCGA_G4_6322	0.00	1.00	0.43	0.94
TCGA_G4_6586	0.85	0.15	0.97	0.43
TCGA_G4_6588	0.85	0.15	0.97	0.43
TCGA_G4_6626	0.86	0.14	0.99	0.39
TCGA_G4_6627	0.00	1.00	0.73	0.93
TCGA_G4_6628	0.86	0.14	0.99	0.40
TCGA_G5_6641	0.00	1.00	0.99	0.91
TCGA_NH_A50T	0.00	1.00	0.50	0.94
TCGA_NH_A50U	0.85	0.15	0.97	0.43
TCGA_NH_A50V	0.85	0.15	0.96	0.44
TCGA_NH_A5IV	1.00	0.00	1.00	0.57
TCGA_NH_A6GA	0.00	1.00	0.61	0.93
TCGA_NH_A6GB	0.72	0.28	0.61	0.74
TCGA_NH_A6GC	0.00	1.00	0.89	0.92
TCGA_NH_A8F7	0.00	1.00	0.43	0.94
TCGA_NH_A8F8	0.87	0.13	1.00	0.39
TCGA_QG_A5YV	0.00	1.00	0.44	0.94
TCGA_QG_A5YW	0.00	1.00	0.59	0.93
TCGA_QG_A5YX	0.00	1.00	0.46	0.94
TCGA_QG_A5Z1	0.00	1.00	0.43	0.94
TCGA_QG_A5Z2	0.00	1.00	0.49	0.94
TCGA_QL_A97D	0.00	1.00	0.65	0.93
TCGA_RU_A8FL	0.79	0.21	0.75	0.66
TCGA_SS_A7HO	0.86	0.14	0.98	0.42
TCGA_T9_A92H	0.00	1.00	0.63	0.93
TCGA_WS_AB45	0.85	0.15	0.97	0.43

Table S8: Results of GSEA associated with high-low C1/C2 ratio

ID	Description	setSize	enrichmentScore	NES	pvalue	p.adjust	qvalues	rank	leading_edge
GO:1903845	negative regulation of cellular response to transforming growth factor beta stimulus	83	0.43	1.56	0.0022	0.0119	0.0061	8447	tags=35%, list=14%, signal=30%
GO:0071559	response to transforming growth factor beta	255	0.30	1.31	0.0047	0.0175	0.0091	8447	tags=29%, list=14%, signal=25%
GO:0030512	negative regulation of transforming growth factor beta receptor signaling pathway	81	0.41	1.51	0.0065	0.0222	0.0115	8447	tags=35%, list=14%, signal=30%
GO:0071560	cellular response to transforming growth factor beta stimulus	249	0.30	1.27	0.0117	0.0339	0.0175	8447	tags=28%, list=14%, signal=24%
GO:1903844	regulation of cellular response to transforming growth factor beta stimulus	122	0.36	1.41	0.0109	0.0323	0.0167	9533	tags=34%, list=16%, signal=29%
GO:0017015	regulation of transforming growth factor beta receptor signaling pathway	120	0.35	1.36	0.0153	0.0412	0.0213	9533	tags=34%, list=16%, signal=29%
